# Impact of Rearing Strategies on the Metabolizable Energy and SID Lysine Partitioning in Pigs Growing from 90 to 200 kg in Body Weight

**DOI:** 10.3390/ani12060689

**Published:** 2022-03-09

**Authors:** Stefano Schiavon, Isaac Hyeladi Malgwi, Diana Giannuzzi, Gianluca Galassi, Luca Rapetti, Paolo Carnier, Veronika Halas, Luigi Gallo

**Affiliations:** 1 Department of Agronomy, Food, Natural Resources, Animals and Environment (DAFNAE), University of Padova, Viale dell’ Università 16, I-35020 Legnaro, Italy; stefano.schiavon@unipd.it (S.S.); luigi.gallo@unipd.it (L.G.); 2 Department of Agricultural and Environmental Sciences—Production, Landscape, Agroenergy (DiSAA), University of Milan, Via G. Celoria 2, I-20133 Milan, Italy; gianluca.galassi@unimi.it (G.G.); luca.rapetti@unimi.it (L.R.); 3 Department of Comparative Biomedicine and Food Science (BCA), University of Padova, Agripolis, Viale dell’Università 16, I-35020 Legnaro, Italy; paolo.carnier@unipd.it; 4 Department of Farm Animal Nutrition, Kaposvár Campus, Hungarian University of Agriculture and Life Sciences, MATE, Guba Sándor Utca 40, H-7400 Kaposvár, Hungary; halas.veronika@uni-mate.hu

**Keywords:** feed restriction, heavy pigs, nutrient partitioning, protein deposition, SID lysine

## Abstract

**Simple Summary:**

The nutritional recommendations for pigs largely focus on pigs with lean genotypes fed ad libitum until reaching up to 140 kg in body weight (BW). Under different rearing conditions, it is still unclear whether existing recommendations apply to pigs that weigh more than 140 kg in BW, especially in heavy pig production systems. In the current study, pigs growing from 90 to 200 kg in BW were raised with different feeding strategies. We observed that energy restriction had a negligible effect on pigs’ estimated metabolizable energy requirements at heavier BW under different feeding conditions. Under energy and protein restrictions, a value of 0.73 could be assumed as the maximum marginal efficiency of standardized ileal digestible lysine (SID lysine) utilization for protein deposition irrespective of BW, which corresponds to 9.8 g of SID lysine per 100 g of protein deposition as a minimum requirement.

**Abstract:**

The current nutrient recommendations focus on pigs fed ad libitum up to 140 kg in body weight (BW). It remains unclear whether this applies to pigs weighing above 140 kg in BW under different rearing conditions. This study aimed to estimate protein (Pd) and lipid (Ld) depositions and the metabolizable energy (ME), standardized ileal digestible lysine (SID lysine) requirement and partitioning in 224 C21 Goland pigs (90–200 kg in BW). The control pigs (C) received diets limiting ME up to 170 kg in slaughter weight (SW) at 9 months of age (SA); older (OA) pigs had restricted diets limiting ME and SID lysine up to 170 kg in SW at >9 months SA; younger (YA) pigs were fed nonlimited amounts of ME and SID lysine up to 170 kg in SW at <9 months SA; and greater weight (GW) pigs were fed as the YA group, with 9 months SA at >170 kg in SW. The estimated MEm averaged 1.03 MJ/kg^0.60^. An 11% increase in MEm was observed in OA pigs compared to the controls. Energy restriction had negligible effects on the estimated MEm. The marginal efficiency of SID lysine utilization for Pd averaged 0.725, corresponding to a SID lysine requirement of 9.8 g/100 g Pd.

## 1. Introduction

Current nutrient recommendations for pigs by the NRC [1] focus on pigs with lean genotypes fed ad libitum until reaching up to 140 kg in body weight (BW). This recommendation has limitations under the management practice(s) of heavy pig production systems for the dry-cured ham industry. For this industry, pigs are fed according to a variety of feeding strategies aimed to manipulate the age (SA) and the weight at slaughter (SW) for the improvement of the ham seasoning aptitude. Such strategies include ad libitum or restricted feeding diets with different energy and amino acid content. A major challenge with these systems is the continuous increase in lean pig genotypes with inadequate ham adiposity for the ham industry, pushing them towards a progressive increase in SW and modifying the feeding strategies [2,3,4]. Recent studies have compared restricted medium-protein diets, restricted low-protein diets and ad libitum high-protein diets for Goland C21 heavy pigs sacrificed at 170 or even at 200 kg in SW, demonstrating that some of these strategies can improve the quality of the green hams [5,6]. However, to optimize the performance of the pigs under such conditions, knowledge of the pigs’ energy and amino acid (AA) requirements and partitioning is important [7,8].

The energy and nutrient utilization of heavy pigs under the dry-cured ham production systems have not been covered by existing literature. It also remains uncertain if the recommended metabolizable energy (ME) requirements for maintenance (MEm = 1.03 MJ/kg in BW^0.60^) by the NRC [1] apply to pigs at heavier BW. Additionally, the nutrient partitioning in heavy pigs kept on feeding strategies with limited or nonlimited energy and/or amino acid supply is yet to be fully addressed. A study by Labussière et al. [9], argued that MEm might not always be independent of ME intake. The energy requirements are a function of feeding level for maintenance requirements and components associated with the BW gain of the pigs. For heavy pigs, whose incidence of MEm in total energy cost was reported to be greater than 45% [10], it is of interest to explore the behaviour of their energy requirements under different rearing conditions and extended ranges of BW. The evaluation of the AA requirements and partitioning of heavy pigs, when kept under different rearing strategies, is critical to nutritional viewpoints. Generally, the AA requirement can be expressed in terms of standardized ileal digestible lysine (SID lysine) when lysine is the first-limiting AA [1]. The SID lysine requirement corresponds to the amount required to achieve the protein deposition (Pd), achievable when the pigs are kept on unlimited feeding and environmental conditions [11,12,13]. The knowledge of the marginal efficiency of SID lysine is necessary to estimate the SID lysine requirement for a given Pd [14]. To achieve this estimate, pigs must be supplied with SID lysine below their requirement for Pd. Additionally, it is crucial to define the potential Pd of the pigs, which can be achieved by not limiting the energy and amino acid supply. According to NRC [1], the marginal efficiency of SID lysine utilization for Pd in heavy pigs is expected to be low, as it would decline with increasing BW. However, this is different from the report of the InraPorc model, which considers the marginal efficiency for SID lysine (0.72) to be constant with increasing BW [15].

The current study aimed to investigate (i) the body protein and lipid accretions of Goland C21 heavy pigs between 90 and 200 kg in body weight (BW) when exposed to various rearing conditions; (ii) the ME and SID lysine partitioning for maintenance and growth and (iii) the marginal efficiency of SID lysine utilization for Pd.

## 2. Materials and Methods

### 2.1. Pig Housing and Rearing

Pigs weighing 95.0 ± 12.5 kg in BW and 149 ± 3 d of age, belonging to the Goland C21 breed (Gorzagri, Fonzaso, Italy) (*n* = 224, barrows and gilts), were divided into 2 batches of 112 pigs as described in [5]. The 2 batches of pigs entered the experimental period sequentially, and tests were conducted during different seasons (autumn–winter and winter-spring). The duration of the experimental period ranged from 85 to 134 d, depending on the rearing strategy. All the pigs from a given batch, born in the same week, were fed the same commercial diets till their transfer to the research pig station of the University of Padua.

An electronic feeding station in each pen (Compident Pig–MLP, Schauer Agrotronic, Prambachkirchen, Austria) was programmed to supply each pig with the planned amount of feed in each pen [16] with 14 pigs per pen (1.57 m^2^/pig). Water was provided ad libitum from nipple drinkers, and the temperature within the room was between 19 and 22 ± 2 °C throughout the experiment. The amount of feed consumed per visit and other feeding behaviour traits were recorded for each pig. The daily dry matter intake (DMI) of each pig was computed from the amount of feed consumed during each visit on that day and its dry matter content.

### 2.2. Live and Postmortem Measurements

Pigs were weighed with an electronic scale at the start and the end of the trial. At each weighing, backfat (BF) depth was measured with an A-mode ultrasonic device (Renco Lean-Meater series 12, Renco Corporation, Minneapolis, MN, USA). The BF measurements were taken from the last rib at approximately 5.5 to 8.0 cm from the midline, at an increasing distance with increasing BW [17]. When pigs reached the average targeted slaughter weight (SW) or age (SA), they were sacrificed following regulations for commercial practices [18].

### 2.3. Experimental Design

Means and standard deviations of the initial BW of the pigs were similar across the pens. A split-plot design with sex within a pen was used. A control group and three other treatment groups, representing 3 alternative rearing strategies, were used. The characteristics of the 4 treatment groups are given in Table 1, from Malgwi et al. [5]. A total of 28 pigs/treatment were assigned to each treatment and housed in two pens. An across-batch rotation scheme was used to assign treatment groups to pens in different batches so that each treatment was assigned to different pens. A description of this procedure is provided below:

(i) The control group (C) had pigs raised under the traditional heavy pig production system for dry-cured ham. Thus, feed restriction was applied and pigs were fed a restricted medium-protein (MP) diet, with lysine as the first-limiting indispensable AA. They were slaughtered at 9 months SA and 170 kg in SW.
(ii) The older pig (OA) strategy was based on a SID lysine restriction in addition to the feed or energy restriction to shift the pigs toward a greater lipid deposition (Ld) and lower Pd to improve the ham seasoning aptitude [19]. Thus, the OA pigs were fed as restrictively as the C pigs, but with feeds lower in SID lysine content (LP). The pigs were slaughtered at >9 months SA at 170 kg in SW. Information from this group of pigs was used to evaluate the ME partitioning and the marginal efficiency of SID lysine utilization for Pd.
(iii) The young pig (YA) rearing strategy set a 170 kg SW target for pigs younger than the minimum age. They were fed a high-protein (HP) diet ad libitum, not limiting indispensable AA content. Such unlimited conditions were applied to exploit the pig potential for Pd and Ld [20,21].
(iv) The third alternative strategy programmed pigs to reach the maximum SW (>170 kg) at 9 months SA (greater weight, GW). The pigs were given the same HP feeds, fed ad libitum, as the YA group and were slaughtered at the same SA (9 months) but at a greater SW (>170 kg) than the C pigs. A comparison between YA and GW was carried out for an evaluation of the effect of an increased SW and SA on energy and SID lysine needs and partitioning at the heavy BW range (170–200 kg in SW) of the pigs.

### 2.4. Feeds

The characteristics of the ingredients used in early diets of pigs 90 to 120 kg in BW and late-finishing diets of pigs >120 kg in BW are given in Table 1. The nutritional composition of the diets is given in Table 2.

**Table 1:** Composition of ingredients in g/kg DM of early diets (90 to 120 kg average BW) and late-finisher diets (over 120 kg in BW).

	Early Finishing Feeds			Late-Finishing Feeds		
Ingredient	High Protein	Medium Protein	Low Protein	High Protein	Medium Protein	Low Protein
Corn grain	350.9	342.0	381.7	388.7	390.2	391.1
Wheat grain	249.5	282.5	272.4	248.4	248.9	249.3
Barley grain	96.4	97.0	97.2	96.9	97.3	97.4
Soybean meal 48% (solv. ex.)	201.0	87.7	39.3	147.3	57.9	18.9
Wheat bran	25.5	84.6	82.6	7.2	55.8	60.7
Wheat middlings	0.0	19.6	29.4	39.1	66.3	88.5
Cane molasses	16.0	16.1	16.3	18.1	18.2	18.2
Lard	22.1	24.0	23.4	22.2	22.3	22.3
Dried-sugar beet pulp	-	9.9	19.8	0.0	9.9	20.4
Calcium carbonate	16.6	16.7	16.7	14.4	14.5	14.5
Dicalcium phosphate	4.8	4.9	4.9	2.2	2.2	2.2
Sodium chloride	3.3	3.3	3.3	3.3	3.3	3.3
Sodium bicarbonate	2.7	2.8	2.8	2.8	2.8	2.8
Vitamin and mineral premix ^a^	2.0	-	2.0	2.0	2.0	2.0
Grapeseed meal	7.3	7.4	7.4	7.4	7.4	7.4
Choline, liquid, 75% ^b^	0.6	0.0	-	-	-	-
L-Lysine ^c^	1.1	1.6	0.7	-	1.1	1.1
DL-Methionine ^d^	0.2	-	-	-	-	-

^a^ Providing per kilogram of feed: vitamin A, 8000 IU; vitamin D_3_, 1200 IU; vitamin E, 8 mg; vitamin B_7_, 0.08 mg; vitamin B_12_, 0.012 mg; niacin, 16.0 mg; biotin, 8 mg; iron, 170 mg; zinc, 117 mg; copper, 14 mg; cobalt, 0.11 mg; iodine, 0.06 mg; manganese, 65 mg; magnesium, 0.14 mg; selenium 10 mg. ^b^ Choline liquid 75% (Methodo Chemicals, 42017 Novellara, RE, Italy). ^c^ L-Lysine Monoclohydrate, 98.5% pure, 78% L-Lysine (Methodo Chemicals, 42017 Novellara, RE, Italy). ^d^ DL Methionine, 98% pure min. (Methodo Chemicals, 42017 Novellara, RE, Italy).

The early finishing HP diets were fed to YA and GW pig groups from 90 to 120 kg in BW. The diet was designed to contain unlimited amounts of SID lysine, methionine, tryptophan and threonine, according to the NRC [1] recommendation for the 70–100 kg in BW range.

The SID lysine content of the early finishing MP feed fed to pigs in the C group was 26% lower than that proposed by NRC [1] for the same BW range. This diet was expected to result in an average daily gain of 0.700 kg/d, with lysine as the first-limiting AA. The SID lysine content of the diet was similar to that frequently used in practice [10].

The SID lysine content of the early finishing LP diet fed to the OA group was consistent, with an average daily gain of 0.650 kg/d. This was purposefully set at a lower amount than that used in previous studies where a shortage of dietary AA content did not influence growth performance, carcass or meat quality [22,23].

The late-finishing HP, MP and LP diets administered from 120 kg in BW onwards were formulated to contain about 20–25% less indispensable SID AA than the corresponding HP, MP and LP feed used in the early finishing period, with lysine as the first-limiting AA.

### 2.5. Feeding Regime

Feeds were administered using the feeding station for individual pigs in all the treatment groups. The restricted amount of feed distributed to the C and OA pig treatments was established based on the average initial BW. Thereafter, the quantity of feed was increased weekly without any further adjustment. The amount of feed administered to C and OA pig groups was increased from 2.3 to 3.0 kg/d throughout the trial, and this corresponds to an increase from 57 to 82 g/kg^0.75^ in metabolic weight, a common practice in such a production system [22].

**Table 2:** Nutrient content (g/kg of DM unless otherwise indicated) of early diets (90 to 120 kg in average BW) and late-finisher diets (over 120 kg in average BW).

	Early Finishing Feeds ^a^			Late-Finishing Feeds		
	HP	MP	LP	HP	MP	LP
Analyzed nutrient composition ^b^						
DM, g/kg as fed	906	904	904	906	902	904
CP (N × 6.25)	178.8	141.6	125.0	152.3	131.9	115.0
Starch	455.8	508.8	539.8	533.1	521.1	542.0
Ether extract	47.5	50.9	48.7	53.0	55.4	53.1
aNDF-NDF	144.6	152.7	156.0	130.2	146.3	148.2
Ash	53.0	52.0	53.1	46.4	45.5	45.4
Lysine (Lys)	9.6	7.3	5.2	7.5	5.5	4.0
Methionine (Met)	3.0	2.4	2.1	2.8	2.2	2.0
Threonine (Thr)	7.2	5.0	4.8	5.5	4.8	3.9
Tryptophan (Trp)	2.0	1.7	1.3	1.4	1.2	1.1
Tyrosine (Tyr)	6.1	4.2	3.8	3.8	3.7	2.9
Calculated nutrient composition ^c^						
ME, MJ/kg DM	14.8	14.6	14.6	14.8	14.6	14.5
NE, MJ/kg DM	11.0	11.1	11.2	11.1	11.1	11.0
CP (N × 6.25)	178.8	141.6	120.6	156.7	128.6	113.9
Digestible CP (DCP)	153.2	120.8	103.2	133.4	109.0	97.0
ME/Digestible CP, MJ/kg DCP	97	121	141	111	134	149
Starch	468.0	496.7	519.9	501.1	521.1	527.7
Linoleic acid	48.6	52.0	52.0	50.8	52.1	52.0
Lys	9.2	6.9	5.0	7.6	5.7	3.9
Met	3.0	2.2	2.0	2.4	2.1	1.9
Thr	6.3	4.8	4.0	5.4	4.2	3.8
Trp	2.2	1.7	1.3	1.9	1.4	1.2
Tyr	5.8	4.5	3.9	5.1	4.1	3.7
SID Lys	8.2	6.0	4.2	6.6	5.0	3.2
SID Met	2.8	2.0	1.8	2.3	1.9	1.8
SID Thr	5.5	4.0	3.2	4.9	3.7	3.1
SID Trp	1.8	1.3	1.0	1.5	1.2	1.0
SID Tyr	5.4	4.1	3.5	4.9	3.9	3.3
Ratios:						
Met/Lys (Optimum = 0.288)	0.34	0.33	0.42	0.35	0.38	0.55
Thr/Lys (Optimum = 0.672)	0.68	0.67	0.76	0.73	0.73	0.97
Trp/Lys (Optimum = 0.182)	0.22	0.22	0.24	0.23	0.24	0.31
Tyr/Lys (Optimum = 0.353)	0.66	0.69	0.84	0.73	0.78	1.03

^a^ HP: high-protein diet, MP: medium-protein diet, and LP low-protein diet. ^b^ Analytical results by averaging data from 4 independent replications. ^c^ Computed according to NRC [1] from the ingredient composition of the feeds (2 batches); SID: standardized ileal digestible amino acid content; optimum ratios according to NRC [1].

### 2.6. Chemical Analysis

Feeds were manufactured by the Progeo Feed Industry (Masone, Reggio Emilia, Italy). Feed samples were collected from the production line and analyzed on the day after collection for evaluation before their use in the experiment to ensure the consistency between the theoretical and actual nutrient contents, with special regard to the AA content [16]. During feed manufacture in the trial and phase feeding, 10 samples of each feed were collected online, pooled, mixed and sampled to obtain a 1 kg feed sample from which independent subsamples were collected. The feed samples were analyzed with 3 independent replications for dry matter (DM: # 934.01; AOAC, 2003), N (# 976.05; AOAC, 2003), ether extract (EE: # 920.29; AOAC, 2003), ash (# 942.05, AOAC, 2003) and neutral detergent fibre with amylase treatment and expressed including residual ash (aNDF) contents [24]. Starch content was determined after hydrolysis of glucose [25] by liquid chromatography [26].

The amino acid content of the feed samples was determined according to the Council of Europe [27,28]. Dietary ME, crude protein, SID amino acid and other nutrient contents were computed from the actual ingredient composition of the feeds and the tabular values for each ingredient [1]. Differences between the analyzed and theoretical AA contents of the feeds were negligible.

### 2.7. Body Composition, Pd and Ld

Body chemical composition was estimated according to Gallo et al. [10], starting from the measurements of BW and BF taken at the start and the end of the experiment. Empty BW (EBW) was estimated from BW using an allometric equation developed for barrows and gilts in the range from 90 to 150 kg in BW, assuming that this equation holds for heavier BW [29]. Body lipid mass (BL) was estimated from BF and BW according to the equation developed by Schiavon et al. [17] using data from different datasets with pigs kept under different feeding conditions over an extended range of BW (12–207 kg in BW). Fat-free EBW mass (FFEBW) was computed as EBW minus BL. Based on the allometric relationships among body protein (BP), water and ash masses [1], BP (kg) was computed as 0.1353 × FFEBW^1.1175^. The Pd and Ld were calculated from the estimated protein and lipid body mass changes throughout the experiment for each treatment. As a control, the backfat and the belly weights collected and measured at slaughter from each pig were regressed against the estimated BL achieved from the BW and BF depth measures taken the day before slaughter.

### 2.8. Metabolizable Energy Partitioning

The energy and lysine partitioning were computed on an individual and daily basis from the estimated changes in body chemical composition and the measured feed intake over the finishing time intervals [10]. The daily ME intake was calculated from the average feed intake and the ME content of diets, adjusted for their actual dietary DM content. The ME used for growth was computed from the estimated Pd and Ld over the trial, assuming a requirement of 44.35 and 52.30 MJ ME/kg of retained protein and lipid, respectively [1]. The amount of ME used for maintenance (MEm) was estimated as ME intake−ME for growth. The resulting MEm value was scaled versus the mean metabolic weight computed as BW^0.60^ [1].

### 2.9. SID Lysine Partitioning

The average SID lysine daily intake was computed from the measured feed intake and the dietary SID lysine content. The SID lysine maintenance requirement for pigs, including that for basal endogenous GIT and integument losses, was computed based on individual feed intake and the average metabolic weight over the testing period [1]. The individual SID lysine retention was computed taking Pd to contain 0.071 lysine, as suggested by the NRC [1]. The SID lysine consumed in excess or the deficit was computed as the difference between SID lysine intake and the estimated requirement for maintenance and Pd. The marginal (above maintenance) SID lysine intake was computed as SID lysine intake minus the SID lysine used for maintenance. The resulting value is expressed per day and per gram of estimated Pd. Total lysine efficiency was estimated as: lysine retained divided by SID lysine intake, while the marginal efficiency of SID lysine utilization for Pd was computed as: lysine retained divided by SID lysine intake minus SID lysine required for maintenance.

### 2.10. Statistical Analysis

Data were analyzed according to the MIXED procedure of SAS (SAS Inst. Inc., Cary, NC, USA) using the following linear model:

y_ijklm_ = µ + treatment_i_ + sex_j_ + (treatment × sex)_ij_ + batch_k_ + pen(treatment × batch)_l:ik_ + ε_ijklm_ (1)

where y_ijklm_ is the observed trait; µ is the overall intercept of the model, treatment is the fixed effect of the i^th^ treatment (i = 1, …, 4); sex_j_ is the fixed effect of the j^th^ sex (j: 1 = barrow, 2 = gilt); (treatment × sex)_ij_ is the interaction effect between treatment and sex; batch_k_ is the random effect of the k^th^ batch (k = 1, 2); pen(treatment × batch)_l:ik_ is the random effect of the lst pen within the interaction treatment × batch, and ε_ijklm_ is the random residual error.

The batch, pen(treatment × batch) and residuals were assumed to be independently and normally distributed with a mean of zero and variances of σ^2^_k_, σ^2^_l_ and σ^2^_m_, respectively. The effect of treatment was tested on the pen(treatment × batch) variance, whereas sex and the treatment × sex interaction were tested on the residual variance.

The 3 degrees of freedom due to the treatment were used to run orthogonal contrasts to test: (1) the C treatment versus the restricted low-protein feeding at the same SW and older SA (OA); (2) the C versus the ad libitum high-protein feeding at the same SW and early SA (YA); (3) the YA vs. GW, representing the influence of an increase in SW (170 vs. 200 kg) and SA (8 vs. 9 mo) under ad libitum high-protein feeding conditions.

## 3. Results

### 3.1. Dry Matter Intake and Growth Performance

The pigs of all the treatment groups, C, OA, YA and GW, were sacrificed after 116, 133, 85 and 116 d of feeding at 265, 282, 234 and 265 d SA, respectively (Table 3). Aside from the GW (185 kg in EBW) group, all pigs were sacrificed at about 164 kg in EBW. 

The protein and energy restriction applied (OA vs. C) induced a reduction in the daily EBW gain (*p* = 0.007) without changing the daily DMI and increased the duration of feeding accompanied by increased cumulative DMI intake (*p* < 0.001). The OA strategy had a negligible impact on the final backfat depth.

The pigs receiving the YA diet had increased daily DMI intake (*p* < 0.001), an EBW gain (*p* < 0.001), final BF depth (*p* = 0.002) and reduced cumulative DMI intake (*p* = 0.006) because of the shorter duration of feeding for the target SW. 

Extending the SW and SA (YA vs. GW), resulted in increased EBW, cumulative DMI and final BF depth (*p* < 0.001) with a reduced daily EBW gain (*p* = 0.021). There was no difference observed in daily DMI.

**Figure 1:**
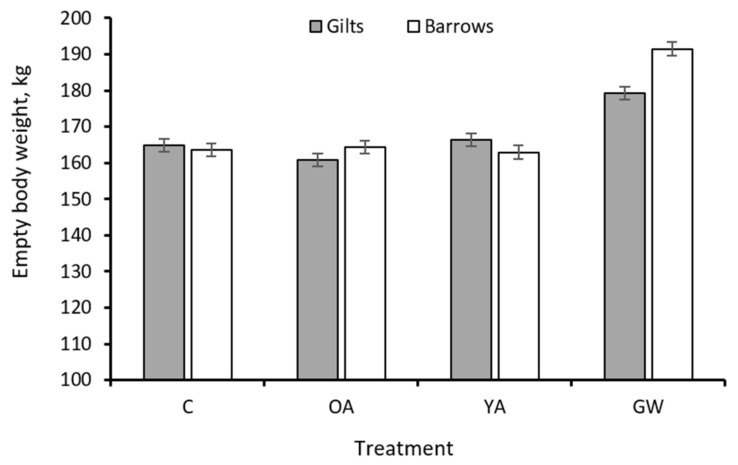
Final empty body weight of C21 Goland barrows and gilts according to different treatments (*n* = 224, sex × treatment interaction *p* = 0.008). C, control pigs fed restricted diets limiting ME supply up to 170 kg in slaughter weight (SW); YA, pigs fed unlimited amounts of ME and protein ad libitum up to 170 kg in SW; GW, pigs fed unlimited amounts of ME and protein ad libitum up to 200 kg in SW; OA, pigs fed restricted diets limiting ME and protein supply up to 170 kg in SW.

**Figure 2:**
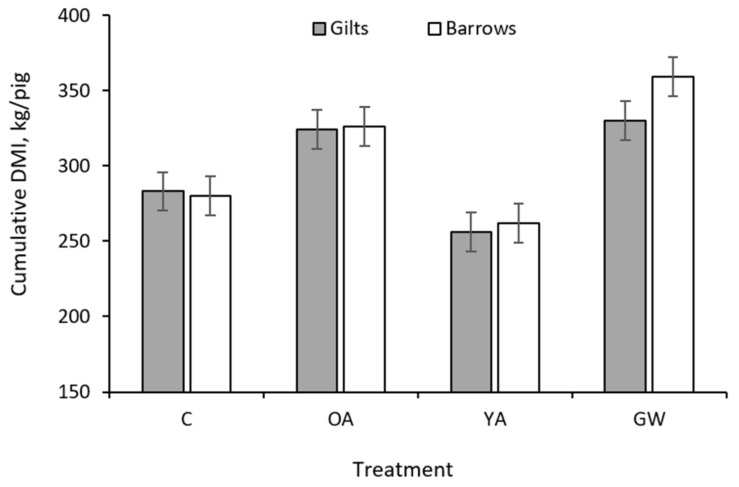
Cumulative dry matter intake of C21 Goland barrows and gilts according to different treatments (*n* = 224, sex × treatment interaction *p* = 0.013). C, control pigs fed restricted diets limiting ME supply up to 170 kg in slaughter weight (SW); YA, pigs fed unlimited amounts of ME and protein ad libitum up to 170 kg in SW; GW, pigs fed unlimited amounts of ME and protein ad libitum up to 200 kg in SW; OA, pigs fed restricted diets limiting ME and protein supply up to 170 kg in SW.

A greater cumulative feed intake (*p* = 0.027) and initial (*p* < 0.001) and final (*p* = 0.008) BF depth was observed in barrows compared to gilts. The sex × treatment interaction was significant for the final EBW (*p* = 0.008) and for the daily (*p* = 0.035) and cumulative DMI intake (*p* = 0.013). The nature of this interaction is such that barrows had a similar final EBW and cumulative DMI for treatment C, OA and YA, but a greater final EBW (Figure 1) and cumulative DMI (Figure 2) only when exposed to the GW treatment.

**Table 3:** Empty body weight (EBW), EBW gain, feed consumption and ultrasound backfat depth of the C21 Goland heavy pigs subjected to different rearing strategies ^1^.

	Treatment					*p* Values			Sex			*p* Values	
Item	C	OA	YA	GW	SEM ^2^	C vs. OA	C vs. YA	YA vs. GW	Gilts	Barrows	SEM ^2^	Sex	Sex × Treatment
Animals, n.	55	56	54	57	-	-	-	-	109	113	-	-	-
Days on feed	116 ± 4	133 ± 8	85 ± 4	116 ± 4	-	-	-	-	114 ± 17	112 ± 19	-	-	-
Empty body weight (EBW), kg													
Initial	84.6	84.1	84.7	85.2	1.31	0.82	0.93	0.80	83.7	85.7	0.96	0.12	0.17
Final ^3^	164.2	162.5	164.7	185.4	1.74	0.52	0.85	<0.001	167.8	170.6	1.23	0.11	0.008
Daily EBW gain, kg/d	0.684	0.589	0.939	0.861	0.02	0.007	<0.001	0.021	0.766	0.770	0.01	0.77	0.20
Feed dry matter intake:													
daily, kg/d	2.42	2.43	3.05	2.96	45.3	0.77	<0.001	0.15	2.68	2.75	32.4	0.079	0.035
Cumulative ^4^, kg/pig	282	325	259	345	12.4	<0.001	0.006	<0.001	298	307	11.9	0.027	0.013
Backfat depth, mm													
initial	10.1	10.1	10.1	10.3	0.30	0.52	0.94	0.95	9.62	10.67	0.30	<0.001	0.85
final	20.8	22.4	24.8	25.9	1.24	0.14	0.002	<0.001	22.83	24.12	1.24	0.008	0.51

^1^ The rearing strategies were as follows: C, pigs fed restricted diets limiting ME supply up to 170 kg in slaughter weight (SW) (fed medium-protein feeds); OA, pigs fed restricted diets limiting ME and protein supply up to 170 kg in SW (fed low-protein feeds); YA, pigs fed unlimited amounts of ME and protein ad libitum up to 170 kg in SW (fed high-protein feeds); GW, pigs fed unlimited amounts of ME and protein ad libitum up to 9 months at slaughter age (about 200 kg in SW) (fed high-protein feeds). ^2^ SEM: pooled standard error of the mean. ^3^ See Figure 1 for the form of the sex × treatment interaction. ^4^ See Figure 2 for the form of the sex × treatment interaction.

### 3.2. Body Composition Changes and ME Partitioning

**Figure 3:**
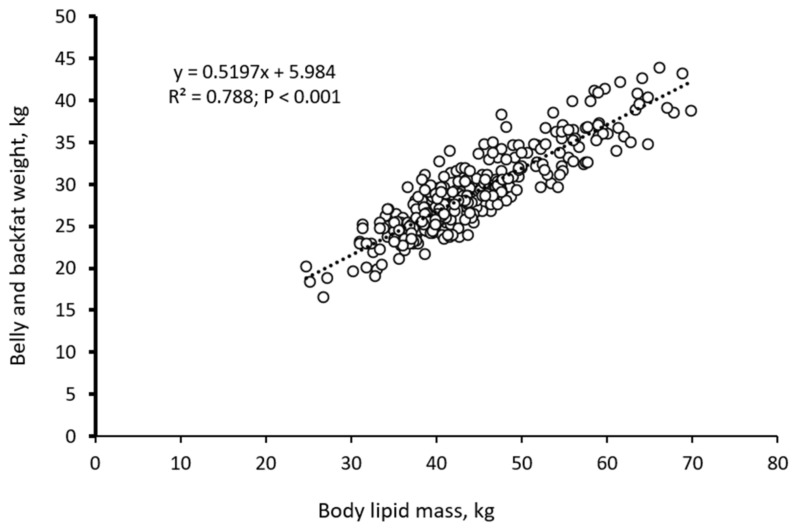
Relation between body lipid mass (×), estimated in vivo from body weight measurements and ultrasound backfat depth the day before slaughtering, and the belly plus backfat weights measured at slaughter (y; *n* = 224).

The weight of the backfat and belly tissues collected at slaughter was linearly correlated (R^2^ = 0.788) to the estimated final body lipid mass (Figure 3). 

The slope of this relationship suggested the backfat plus the belly weight represented about 0.52 of whole-body lipid mass.

The OA treatment had no influence on the estimated final body lipid mass and Ld, compared to C (Table 4), but it reduced Pd for growth compared to C by 7% (*p* < 0.001). Nevertheless, OA treatment resulted in a similar final body protein mass as that achieved from the C treatment because the OA pigs had a greater number of days on feed. Despite the same ME intake, the OA pigs utilized 17% less ME for Pd (*p* < 0.001) and 11% more for maintenance (*p* < 0.001) than the pigs receiving a C diet.

The YA pigs presented a greater final body lipid mass (12%, *p* = 0.010), Ld (64%, *p* < 0.001) and Pd (24%, *p* < 0.001), but a 4.4% lower final whole-body protein mass (*p* = 0.014) compared to the C pigs. This lower estimated protein mass was due to earlier attainment of the targeted SW by the YA pigs. The ME intake and ME utilized for growth (Pd and Ld) were 28 and 52% greater in YA compared to C pigs (*p* < 0.001), respectively. However, the ME for maintenance for a unit of MW remained unchanged for the pigs undergoing the two treatments.

The estimated final whole-body lipid and protein masses of GW pigs were greater (*p* < 0.001) than the corresponding masses of the YA pigs as a result of increased SW and SA. The Ld and Pd of the GW pigs tended to be 7–11% lower (*p* < 0.07 and 0.06, respectively) than the corresponding values of the YA pigs, reflecting the decline in the growth impulse with advancing SW and SA. Compared to YA, the GW pigs had similar daily ME intake with 10% less ME dedicated to growth, and the MEm remained the same, despite the remarkable increase in SW.

**Figure 4:**
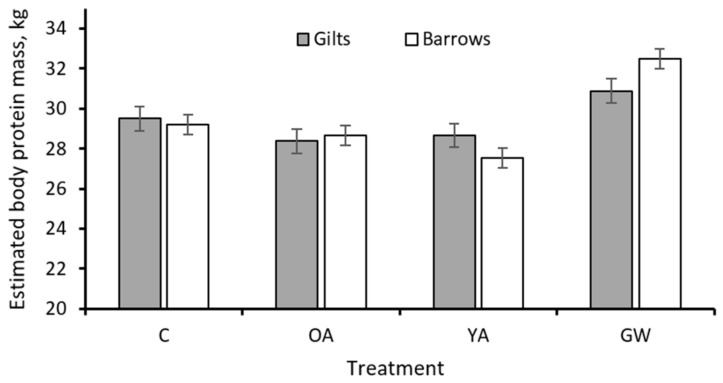
Estimated final body protein mass of C21 Goland barrows and gilts according to different treatments (*n* = 224, sex × treatment interaction *p* = 0.009). C, control pigs fed restricted diets limiting ME supply up to 170 kg in slaughter weight (SW); YA, pigs fed unlimited amounts of ME and protein ad libitum up to 170 kg in SW; GW, pigs fed unlimited amounts of ME and protein ad libitum up to 200 kg in SW; OA, pigs fed restricted diets limiting ME and protein supply up to 170 kg in SW.

**Figure 5:**
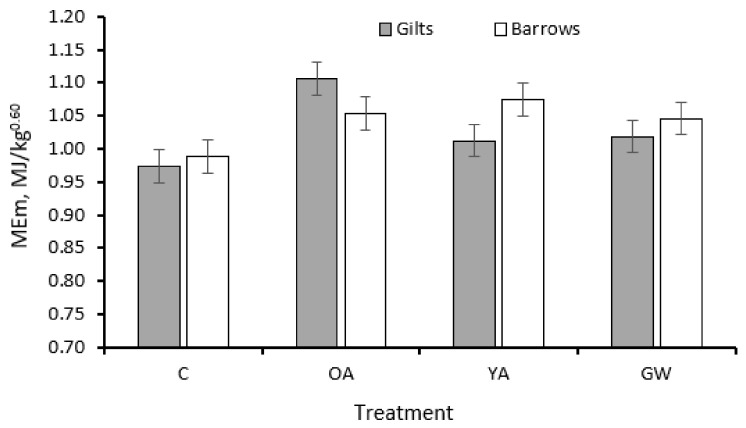
Estimated metabolizable energy used for maintenance (MEm) of C21 Goland barrows and gilts according to different treatments (*n* = 224, sex × treatment interaction *p* = 0.001). C, control pigs fed restricted diets limiting ME supply up to 170 kg in slaughter weight (SW); YA, pigs fed unlimited amounts of ME and protein ad libitum up to 170 kg in SW; GW, pigs fed unlimited amounts of ME and protein ad libitum up to 200 kg in SW; OA, pigs fed restricted diets limiting ME and protein supply up to 170 kg in SW.

The estimated initial (*p* = 0.005) and final (*p* = 0.012) lipid masses were 5–7% greater in barrows compared to gilts, but no differences were observed for the initial and final protein masses. Barrows and gilts had similar ME partitioning between growth and maintenance. Significant sex × treatment interactions were observed for the final estimated whole-body protein mass (*p* = 0.009; Figure 4) and the ME intake (*p* = 0.035) because the barrows responded differently from gilts only when subjected to the GW treatment. There was significant sex × treatment interaction for the estimated MEm (*p* = 0.001; Figure 5). However, the observed differences between the barrows and the gilts among all treatments for MEm were of small magnitude.

**Table 4:** Estimated body composition changes and metabolizable energy (ME) partitioning of the C21 Goland heavy pigs subjected to different rearing strategies ^1^.

	Rearing Strategy					*p* Values			Sex			*p* Values	
Item	C	OA	YA	GW	SEM ^2^	C vs. OA	C vs. YA	YA vs. GW	Gilts	Barrows	SEM	Sex	Sex × Treatment
Estimated body lipid mass ^3^, kg													
initial	14.6	14.5	14.6	14.8	0.39	0.87	0.97	0.62	14.1	15.1	0.30	0.005	0.44
final	41.0	42.5	46.1	53.4	1.63	0.37	0.010	0.001	44.6	47.0	1.38	0.012	0.13
daily lipid deposition (Ld), g/d	226.4	209.8	370.1	331.4	14.2	0.40	<0.001	0.07	278.4	290.5	9.01	0.13	0.26
Estimated body protein mass ^4^, kg													
initial	15.6	15.5	15.6	15.7	0.24	0.80	0.80	0.88	15.5	15.7	0.17	0.30	0.11
final	29.4	28.5	28.1	31.7	0.46	0.08	0.014	<0.001	29.4	29.5	0.40	0.71	0.009
daily protein deposition (Pd), g/d	118.2	97.7	146.5	137.5	6.16	0.001	<0.001	0.06	0.13	0.12	5.67	0.39	0.33
Energy balance:													
ME intake ^5^, MJ/d	35.3	35.5	45.1	43.7	0.67	0.86	<0.001	0.15	39.4	40.4	0.47	0.08	0.035
ME for growth ^6^, MJ/d	17.0	15.2	25.9	23.4	0.74	0.12	<0.001	0.039	20.2	20.7	0.43	0.23	0.19
for Pd	5.2	4.3	6.5	6.1	0.27	<0.001	<0.001	0.06	5.6	5.5	0.25	0.39	0.34
for Ld	11.8	10.9	19.4	17.3	0.74	0.40	<0.001	0.07	14.6	15.2	0.47	0.13	0.26
ME for maintenance ^7^, MJ/kg^0.60^	0.981	1.091	1.029	1.036	0.021	<0.001	0.08	0.79	1.02	1.04	0.015	0.34	0.001

^1^ The rearing strategies were as follows: C, control pigs fed restricted diets limiting ME supply up to 170 kg in slaughter weight (SW); YA, pigs fed unlimited amounts of ME and protein ad libitum up to 170 kg in SW; GW, pigs fed unlimited amounts of ME and protein ad libitum up to 200 kg in SW; OA, pigs fed restricted diets limiting ME and protein supply up to 170 kg in SW. ^2^ SEM: pooled standard error of the mean. ^3^ Estimated from empty BW and backfat thickness according to Schiavon et al. [17]. ^4^ Estimated from fat-free empty BW (FFEBW = EBW—body lipid) using allometric relationships of body protein with body water and ash (NRC [1]). See Figure 4 for the form of the sex × treatment interaction for the final body protein mass. ^5^ Computed from measured feed intake (FI) and tabulated ME content of feed ingredients (NRC [1]). The form of the sex × treatment interaction was similar to that of DMI given in Figure 1. ^6^ Computed assuming a requirement of 44.4 and 52.3 MJ/kg of protein and lipid retained, respectively (NRC [1]). ^7^ ME used for maintenance computed as (ME intake—ME for Ld—ME for Pd)/average metabolic weight.

### 3.3. SID Lysine Partitioning and Efficiencies of Utilization

Despite the DMI being the same between the OA and C treatments, the former reduced the total (*p* = 0.007) and the daily marginal SID lysine intake (*p* = 0.006) compared to the OA treatment due to low level of dietary SID lysine in the LP feeds (Table 5). However, when expressed as per gram of estimated Pd, the marginal SID lysine intake of pigs fed OA was similar to that of the C pigs. All the pigs undergoing these two treatments had an SID lysine shortage compared to their estimated requirement. The estimated SID lysine requirement for Pd of the OA group was about 17% lower than that for the C group (*p* < 0.001) due to the SID lysine restriction in the LP diet. The marginal efficiency of SID lysine utilization for Pd increased significantly by 11% (*p* = 0.009) as a consequence of the SID lysine restriction in the OA compared to C treatment.

Compared to the C treatment, total SID lysine and marginal intakes were higher (*p* < 0.001) in the YA pigs with SID lysine remarkably above the estimated requirements. Thus, the total and the marginal efficiencies of SID lysine utilization for Pd of the YA pigs were much lower (27%) compared to the C pigs (*p* < 0.001).

**Figure 6:**
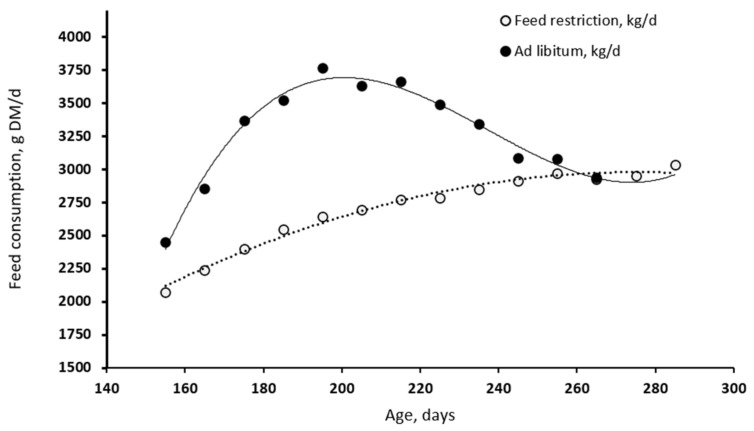
Daily feed DM consumption of the C21 Goland pigs fed ad libitum or restricted with increasing days of age. Each point represents a mean of 1176 to 1456 individual daily observations (*n* = 224).

Differences between YA and GW pigs for the SID lysine partitioning were, in general, not significant or negligible, except for the daily SID lysine marginal intake (*p* = 0.05). The lower SID lysine marginal intake of the GW group was in part due to the reduction in the daily DMI during the last part of the finishing period (Figure 6) and to the increased average metabolic weight over the extended late-finishing period.

Compared to the barrows, gilts had greater SID lysine marginal intake, both when expressed in absolute terms (*p* = 0.049) and per gram of Pd (*p* = 0.002). Conversely, the gilts showed a slightly lower total (*p* = 0.007) and the marginal efficiency of SID lysine utilization for Pd compared to barrows (*p* = 0.032). Significant sex × treatment interaction was observed for the SID lysine intakes (*p* = 0.007) and daily marginal intakes (*p* = 0.041), due to the higher DMI of the barrows in the GW treatment compared to gilts. The form of these interactions was similar to that observed for DMI (Figure 1), but the magnitude of the differences was negligible.

## 4. Discussion

### 4.1. Growth Performance and Dry Matter

We recently proposed that alternative rearing strategies could offer benefits in terms of growth performance and ham quality compared to the traditional C treatment [5]. The reader is invited to refer to this companion paper for a detailed discussion about the implication of the various rearing strategies on the growth performance, carcass and ham quality of the pigs under investigation [5]. However, it is important to mention in the current paper that:

(i) The range of BW studied is much heavier than that commonly practised in fresh meat production. Note, for instance, that nutrient recommendations for growing pigs heavier than 140 kg in BW are not currently available [1,30].
(ii) With the current C feeding regime, the degree of DMI or energy restriction was remarkable and in the order of 20%, similar to what is practised in some regions of Spain for dry-cured ham production [31].
(iii) A rearing practice based on protein restriction, in addition to the energy restriction, decreased the daily EBW gain and increased the duration of feeding for the target SW, but it had a small influence on the in vivo backfat depth compared to the C treatment.
(iv) C21 Goland pigs receiving the YA treatment evidenced a remarkable increase in EBW gain (0.939 kg/d) and backfat depth compared to the C treatment, despite the heavy range of BW (90–170 kg). Differences among individual pigs cannot be fully expressed when the pigs are kept on a restricted feeding regime, as the major factor limiting the growth is the energy and nutrient supply [32]. Therefore, the response of an EBW gain when shifting pigs from a restricted to ad libitum feeding strategy would largely depend on the growth characteristics of the pig genotype used [21]. This implies that moving from a restricted to voluntary feeding regime would lead to a greater heterogeneity among pigs intended for dry-cured ham production [23].
(v) Data from the current experiment evidenced that the voluntary DMI of the YA and GW pigs peaked at 3.750 kg/d at about 190 d of age, with a decline of 11.9 g/d (0.17 MJ/d of ME) thereafter. Assuming that voluntary feed intake is determined by the pig’s attempt to fulfil its energy demands [33], the decline would represent the progressive decrease in the ME demand for growth with increasing maturity. However, this is partially counterbalanced by an increase in the demand for MEm.
(vi) Sex had some influence on growth performance. The barrows had greater cumulative DMI (+3.0%) and final backfat depth (+5.7%) but similar EBW gain compared to gilts. This suggests that barrows had a greater propensity for body fatness than gilts, as is in agreement with previous literature [34,35]. Similarly, few differences between barrows and gilts were observed in the same breed of pigs in a different study [10]. In our current work, we expected a greater difference between sex, because the ad libitum feeding regime would exploit the propensity for the growth of the various body parameters measured. However, this expectation was evidenced between barrows and gilts under the GW but not YA strategy. This is attributed to the effect of greater SW and SA resulting from the GW treatment, thus, suggesting that the differentiation between barrows and gilts would become more evident after 8 months of age and >170 kg in BW under unlimited feeding conditions.

### 4.2. Chemical Body Composition Estimates

The knowledge of energy and nutrient intake and body chemical composition changes is essential for estimating nutrient partitioning and requirements. Nutritional recommendations for maintenance are commonly based on the knowledge of metabolic weight or the body protein mass, while those for growth are based on Pd and Ld [1,21,36]. Recommendations have been developed based on comparative slaughter experiments with groups of pigs slaughtered at different ages [37,38]. Under practical conditions, the slaughtering of pigs is not feasible, for reasons such as time and costs. In pigs, reasonable estimates of body composition can be achieved from measurements of BW and BF depth [17,39]. This is achieved through repeated measurements on individual pigs from a given population. Earlier, it was proposed that the allometric relationships between body components (such as body water, body protein, body ash, etc.) are easily computed once the BL is estimated [1,20]. However, it is crucial to note that good estimates of body composition are dependent on the accuracy and precision of the equation that estimates the BL from BW and BF depth measurements. This is due to the variability between equations proposed by different researchers with respect to the difference in pig genotype, BW range, feeding conditions, environment and so on. For instance, an equation for BL estimation proposed by Kloareg et al. [29] suggests that for each mm of BF depth increase, the BL percentage increases by 0.0113, while that of Schiavon et al. [17], suggests that the BL percentage increases by 0.007 for each mm increase in BF depth. Such discrepancies lead to strong differences in the estimation of body composition. In the current experiment, we used the equation proposed by Schiavon et al. [17] developed using data from comparative slaughter experiments conducted in the UK and Italy on pigs of different genotypes and sexes, with BW ranging from 12 to 200 kg fed ad libitum or with restricted feeds differing in nutrient contents. Our current results indicate that the estimated BL mass was linearly correlated to the weights of the fat tissues at slaughter (R^2^ = 0.788). Additionally, the estimated MEm and the SID lysine maximum marginal efficiency of utilization for Pd obtained in the current paper are consistent with the values obtained by other authors [1,15]. Therefore, this suggests that the procedure adopted in the current paper for estimation of body composition changes was reliable for practical application.

Among treatments, the estimated final body protein mass ranged from 28.1 to 31.7 kg. A protein mass ranging from 32 to over 50 kg has been suggested for mature pigs, with the highest values for pigs belonging to the nucleus of improved genotypes [21,40]. Compared to the C treatment, the OA treatment had no impact on the estimated final body protein mass, despite the lower daily Pd, as the pigs of this treatment had more time to complete their protein growth. Conversely, when exposed to the YA conditions, the pigs accumulated 4.4% less final body protein mass compared to the C treatment, despite the greater daily Pd, because of the shorter duration on feed. Results also indicate that the C21 Goland pigs at 170 kg in SW and 8 months of age (YA) had not reached their mature body protein mass, as when kept on feed for one additional month (GW vs. YA), they demonstrated an increase in body protein mass of 13% and Pd in the order of 116 g/d.

The various treatments had a strong impact on the estimated final whole-body lipid mass, with values in the order of 41–43 kg for C and OA pigs and 46.1–53.4 kg for YA and GW pigs. The body fatness status, expressed as the ratio between the final lipid and protein masses, ranged between 1.4–1.5 and 1.6–1.7 for the C and OA and the YA and GW groups, respectively. For the ham-producing industry, the greater body fatness status of the latter groups is an indication that ad libitum feeding can increase their profitability and reduce the incidence of pigs that are too lean at slaughter, which does not meet the quality standards [2].

When provided with low essential dietary AA contents, pigs balance their nutrient demands by increasing their feed intake to meet their potential for Pd [16]. Thus, an increased feed intake causes an extra amount of energy intake, which results in an extra fat deposition. However, this does not necessarily reflect the pigs’ genotypic characteristics for fat deposition, but rather the interaction between the pigs’ genotype, the energy density and the AA-to-energy ratio of the diet. In contrast, under unlimited energy and AA supply, pigs can express their potential for both Pd and Ld [11,12]. In our current experiment, the HP diets were formulated to be unlimited in energy and AA, and we intended to stimulate the Goland C21 pigs under YA and GW treatments to express their potential for both Pd and Ld, without the confounding effects related to the energy and AA densities of the feeds [18,21]. Thus, the voluntary DMI and the resulting final lipid-to-protein ratio achieved by the YA and GW pigs in the current experiment would represent the desire of the C21 Goland pigs to attain a given body fatness status, with modest influences due to the characteristics of the feeding resource [11,12].

### 4.3. Estimated Daily Protein Depositions

Experiments conducted with heavy pigs under conventional feeding regimes revealed estimated Pd in the order of 100 ± 20 g/d [41,42,43], in agreement with the estimates (118 g/d) achieved for the C treatment in the present study. Previous studies using heavy pigs (80–170 kg in BW) of various genotypes fed restricted diets have repeatedly investigated the effects of the reduction in SID lysine levels up to 4.8 and 3.5 g/kg when fed in early and late-finishing periods, respectively. These reductions were found not to influence growth performance, carcass quality or the dry curing aptitude of the fresh hams, and these studies failed to evidence an effect on Pd [10,22,44]. In the present study, the C dietary SID lysine densities were kept at 6.0 and 5.0 g/kg DM (5.4 and 4.5 g/kg as fed) in the early and late-finishing periods, respectively. This suggests that the SID lysine supplied in the C diet would have been adequate. On the other hand, the restricted feeding conditions could have limited the Pd of the pigs. Some of the literature indicated that even with an adequate AA supply, the partitioning of dietary ME between Pd and Ld could be influenced by the ME [37]. This issue is debated, and discrepancies remain about the actual influence of ME supply on Pd and Ld in the existing literature. For instance, the model proposed by InraPorc assumes that the response of Pd to the ME supply follows a curvilinear plateau function [15], while NRC [1] suggested a linear plateau, with slopes that decrease with increasing BW. In our current experiment, we found that the C treatment induced a 19% reduction in Pd (119 g/d) compared to 147 g/d when YA was provided. In our C-fed pigs, the SID lysine marginal intake averaged 0.110 g/g Pd. This value was lower than the 0.125 g/g Pd requirement suggested by NRC [1] for pigs 120 kg in BW. This might suggest that in our current experiment the Pd of the C pigs was primarily reduced as a consequence of the inadequate SID lysine supply, but this does not exclude the possibility that a restricted ME intake could have limited Pd in pigs subjected to an energy restriction.

The OA pigs had similar DMI as C pigs with a remarkable reduction in Pd (119 to 97 g/d) which could be entirely attributed to the low SID lysine supply with the LP feeds (4.2 and 3.2 g/kg DM in the early and late-finishing period, respectively) compared to the MP. This restriction resulted in further marginal SID lysine intake reduction from 0.110 to 0.098 g/g Pd. This outcome is consistent with Wecke and Liebert [45] who reported a need for 0.100 g/g Pd of SID lysine marginal intake for pigs 15 to 110 kg in BW, despite the BW difference in our current investigation (90 to 170 kg in BW).

The estimated Pd of the YA pigs averaged 147 g/d, similar to previous studies conducted on modern pig genotypes, but for lighter BW ranges [21,45]. As the pigs were managed under unlimited, ambient feeding and health conditions, we could infer that the obtained value of 147 g/d might be close to the actual potential Pd of the C21 Goland genotype. According to NRC [1] and other studies [21,45], the point of maximum Pd, about 150 g/d, falls between 50 and 75 kg in BW, such that the pigs of our current experiment 90 to 200 kg in BW fell in the region of declining Pd. Extending the SA with GW, the final body protein mass increased by about 12.8%, but the daily Pd was slightly reduced (5.5%) compared to YA. Such a declining trend in Pd with increasing slaughter BW/age is consistent with pre-existing observations that the Pd rate decreases with increasing age after a point of maximum Pd, up to the attainment of mature-body protein mass [11,12,20].

Overall, the Pd estimates achieved in the present study suggest that the Goland C 21 pig genotype has good potential for Pd even at heavy BW, as is expected for modern pig genotypes. 

### 4.4. Metabolizable Energy Requirements and Partitioning

The estimated MEm requirement of the pigs of our current study averaged 1.03 MJ/kg^0.60^, in agreement with the value of 1.02 MJ/kg^0.60^ proposed by Noblet et al. [46], although some variation across different genotypes and sexes were expected. Similarly, Milgen et al. [47] suggested that the fasting heat production of lean pigs is close to 0.962 MJ/kg^0.60^, or slightly more because of physical activity and thermoregulation. NRC [1] suggests a standard maintenance requirement of 0.824 MJ/kg^0.60^, with additional energy for thermogenesis, increased physical activity and genotype adjustment. Estimation of MEm, as a difference between ME intake and the ME used for growth, is influenced by the estimated Pd and Ld, and thus by the assumed ME efficiency for protein (kp) and lipid (kl) deposition. The values of kp and kl found by Noblet et al. [46] were 0.62 and 0.84, respectively. In the current study, the lower kp and kl values used, 0.53 and 0.75, were selected according to NRC [1]. The use of these coefficients implies that the calculated MEm values of the current research could have been greater than the values achievable using the Noblet et al. [46] partial efficiencies. On the other hand, NRC [1] also reported that kp and kl can considerably vary from 0.36 to 0.57 and 0.57 to 0.81, respectively. Therefore, despite the heavier weight of pigs in our present study, we achieved an MEm estimate consistent with existing research in the literature. This suggests that our MEm estimates can be applied for heavier BW (>170 kg), which were not accounted for by the NRC [1].

Furthermore, there was an indication that MEm may increase under the condition of energy and protein restriction (OA compared to C). To our knowledge, no existing pieces of evidence reported that such a restriction strategy would increase the MEm requirement. However, such a result must be treated with caution, as the chemical body masses of the pigs in this current research were estimated from simple body measurements of BW and BF depth. Some evidence indicates that diets with insufficient indispensable AA contents increase intramuscular fat with little influence on the BF depth [48]. For this reason, a greater MEm utilization in the OA pigs in the current research could also be attributed to an increased intramuscular fat that was not entirely captured by the simple body measurements of BW and BF depth. On the other hand, our current research suggests that the need for MEm is not influenced by feed (energy) restriction or by increased SW. We can also infer that the NRC [1] MEm requirement of 1.02 MJ/kg^0.60^ is applicable for pigs weighing more than 140 kg in BW regardless of the restricted feeding regime.

No relevant differences were evidenced between barrows and gilts in body composition changes in terms of Pd and ME intake and ME partitioning. This agreed with our previous studies [23], where little influence from sex or sex interaction on the partitioning of ME intake towards Pd, Ld and maintenance was observed.

### 4.5. SID Lysine Partitioning and Efficiency

According to NRC [1], deposited protein contains 7.1% lysine, and for maintenance, the efficiency of lysine utilization is 0.75 while the maximum efficiency of SID lysine utilization for Pd declines with BW from 0.682 (20 kg in BW) to 0.568 (120 kg in BW). These efficiencies are equivalent to a requirement of 10.4 and 12.5 g SID lysine per 100 g of Pd at 20 and 120 kg in BW, respectively. Dourmad et al. [49] investigated pigs fed ad libitum from 50 to 110 kg in BW and reported that the marginal efficiency of SID lysine utilization ranged between 0.65 and 0.70, which is equivalent to a requirement of 10.0–10.9 g/100 g Pd. Similarly, Wecke and Liebert [45] reported that 17–18 g/d SID lysine was required for 170 g/d Pd.

In our current study, the YA and the GW pigs consumed SID lysine well in excess compared to the estimated requirement based on the NRC [1] equation. Thus, the estimated total and marginal efficiency of SID lysine utilization for Pd is of little biological significance, and no comparison with the other treatments can be discussed. In contrast, it was estimated that the SID lysine intake of both the C and the OA pigs was below the estimated requirements. Under such conditions, the marginal efficiency of SID lysine utilization for Pd reflects the efficiency of the pig to utilize the SID lysine in protein accretion. The C pigs fed a restricted medium-protein diet evidenced a marginal efficiency of SID lysine utilization of 0.650. This corresponds to an SID lysine requirement of about 10.9 g/100 g of Pd. Similarly, the marginal efficiency of SID lysine utilization for Pd of the OA pigs averaged 0.725, which corresponds to a requirement of 9.8 g/100 g Pd. This value is similar to that proposed by the InraPorc model, which is assumed to be constant throughout growth [15]. In contrast, NRC [1] reported that the marginal efficiency of SID lysine utilization for Pd declines with increasing BW. On the contrary, the results we obtained in the current experiment suggest that the marginal efficiency of SID lysine utilization for Pd does not change with increasing BW, in agreement with the InraPorc model [15]. This information is valuable in defining feeding strategies and formulating diets optimized in terms of AA supply according to the desired growth and the optimal body fatness statuses at slaughter for heavy pigs. Regarding the effect of sex, no relevant difference in the marginal efficiency of SID lysine utilization for Pd was observed.

## 5. Conclusions

The modelling approach, based on repeated BW and BF measurements, used in the current experiment could have a practical application in estimating the ME and the amino acid requirement of growing pigs through extended BW and feeding conditions. We found that the energy restriction had little or no influence on the estimated MEm. This study also confirmed that an MEm value of 1.02 MJ/kg^0.60^ is applicable for pigs weighing 90 to 200 kg in BW, irrespective of the feeding regime. Our results suggest that under energy and protein restriction, the maximum marginal efficiency of SID lysine utilization for protein deposition (Pd) was 0.73. This corresponds to 9.8 g of SID lysine per 100 g of Pd, as a minimum requirement, irrespective of body weight (BW).

## Figures and Tables

**Table 5:** Lysine partitioning and efficiencies of standardized ileal digestible (SID) lysine utilization of the C21 Goland heavy pigs subjected to different rearing strategies ^1^.

	Feeding Strategy					*p* Values			Sex			*p* Value	
Item	C	OA	YA	GW	SEM ^1^	C vs. OA	C vs. YA	YA vs. GW	Gilts	Barrows	SEM	Sex	Sex × Treatment
SID lysine intake ^2^, g/d	14.6	11.3	24.3	23.0	0.70	0.007	<0.001	0.21	18.0	18.6	0.38	0.05	0.007
SID lysine marginal intake ^2^,													
per day, g/d	12.9	9.6	22.3	21.0	0.68	0.006	<0.001	0.05	16.2	16.7	0.37	0.049	0.041
per gram of protein deposited, g/g	0.110	0.099	0.154	0.155	0.009	0.14	<0.001	0.89	0.126	0.133	0.008	0.002	0.022
SID lysine consumed in excess ^3^, g/d	−1.48	−2.26	4.36	4.19	1.14	0.35	<0.001	0.84	0.85	1.55	1.03	0.002	0.21
Lysine losses and retention ^4^, g/d:													
basal GIT losses	1.08	1.09	1.36	1.32	0.02	0.77	<0.001	0.15	1.20	1.23	0.01	0.08	0.035
integumental losses	0.17	0.17	0.17	0.19	0.001	0.51	0.81	<0.001	0.18	0.18	0.001	0.07	0.51
retained	8.39	6.94	10.40	9.76	0.44	<0.001	<0.001	0.06	8.94	8.81	0.40	0.39	0.34
SID lysine requirement ^5^, g/d:													
maintenance	1.67	1.68	2.05	2.01	0.03	0.81	<0.001	0.31	1.83	1.87	0.02	0.07	0.029
protein deposition (Pd)	14.37	11.86	17.92	16.81	0.77	<0.001	<0.001	0.06	15.32	15.16	0.71	0.53	0.18
total	16.04	13.54	18.82	19.97	0.76	<0.001	<0.001	0.06	17.16	17.03	0.67	0.65	0.15
SID lysine efficiencies ^6^:													
total efficiency	0.576	0.616	0.432	0.428	0.024	0.050	<0.001	0.83	0.522	0.504	0.02	0.007	0.81
marginal efficiency	0.650	0.725	0.472	0.469	0.025	0.009	<0.001	0.91	0.566	0.538	0.016	0.032	0.66

^1^ The rearing strategies were as follows: C, control pigs fed restricted diets limiting ME supply up to 170 kg in slaughter weight (SW); YA, pigs fed unlimited amounts of ME and protein ad libitum up to 170 kg in SW; GW, pigs fed unlimited amounts of ME and protein ad libitum up to 200 kg in SW; OA, pigs fed restricted diets limiting ME and protein supply up to 170 kg in SW. SEM: pooled standard error of the mean. ^2^ SID lysine computed from feed intake and dietary SID lysine content (NRC [1]). The form of the sex × treatment interaction was similar to that of DMI given in Figure 1. Marginal intakes were computed as SID lysine intake—SID lysine requirement for maintenance (NRC, 2012). The resulting amount was as expressed per day and per gram of estimated protein deposition. ^3^ SID lysine consumed in excess of the requirement was computed as SID lysine intake − the SID lysine requirement for maintenance and protein deposition (NRC [1]). ^4^ Basal gastrointestinal and integumental losses of lysine were estimated from dry matter intake and metabolic weight BW^0.75^), as indicated by NRC [1]. The form of the sex × treatment interaction was similar to that of DMI given in Figure 1. However, the magnitude of the differences was negligible. SID lysine retained was assumed to be 0.071 of protein gain (NRC [1]). ^5^ SID lysine requirements for maintenance and protein gain were computed according to NRC [1]. The form of the sex × treatment interaction was similar to that of DMI given in Figure 1. However, the magnitude of the differences was negligible. ^6^ Total efficiency was computed as lysine retained/SID lysine intake. Marginal efficiency was computed as lysine retained/SID lysine marginal intake.

## Data Availability

The data supporting the findings of this study are available from Gorzagri s.s., but restrictions apply to the availability of these data, which were used under license for the current study and are not publicly available. Data are, however, available from the authors upon reasonable request and with permission from of Gorzagri s.s.
